# A Review of the Antiviral Susceptibility of Human and Avian Influenza Viruses over the Last Decade

**DOI:** 10.1155/2014/430629

**Published:** 2014-04-02

**Authors:** Ding Yuan Oh, Aeron C. Hurt

**Affiliations:** ^1^WHO Collaborating Centre for Reference and Research on Influenza, 10 Wreckyn Street, North Melbourne, VIC 3051, Australia; ^2^School of Applied Sciences and Engineering, Monash University, Churchill, VIC 3842, Australia

## Abstract

Antivirals play an important role in the prevention and treatment of influenza infections, particularly in high-risk or severely ill patients. Two classes of influenza antivirals have been available in many countries over the last decade (2004–2013), the adamantanes and the neuraminidase inhibitors (NAIs). During this period, widespread adamantane resistance has developed in circulating influenza viruses rendering these drugs useless, resulting in the reliance on the most widely available NAI, oseltamivir. However, the emergence of oseltamivir-resistant seasonal A(H1N1) viruses in 2008 demonstrated that NAI-resistant viruses could also emerge and spread globally in a similar manner to that seen for adamantane-resistant viruses. Previously, it was believed that NAI-resistant viruses had compromised replication and/or transmission. Fortunately, in 2013, the majority of circulating human influenza viruses remain sensitive to all of the NAIs, but significant work by our laboratory and others is now underway to understand what enables NAI-resistant viruses to retain the capacity to replicate and transmit. In this review, we describe how the susceptibility of circulating human and avian influenza viruses has changed over the last ten years and describe some research studies that aim to understand how NAI-resistant human and avian influenza viruses may emerge in the future.

## 1. Background

### 1.1. Influenza: The Disease and the Virus

Influenza is a highly contagious, respiratory disease that is primarily transmitted via airborne contact with virus-laden secretions from an infected person. Typical symptoms range from fever, malaise, sore throat, and muscular pain to fatal pulmonary or cardiac complications, often due to primary viral or secondary bacterial infections [1]. Most influenza infections are self-limiting, lasting for one to five days without further complications, but host factors such as age, pregnancy, smoking, and underlying medical conditions can increase the severity of illness [2].

Influenza viruses are members of the Orthomyxoviridae, a family of enveloped negative sense, single-stranded ribonucleic acid (RNA) viruses with segmented genomes [3]. Two surface glycoproteins exist on the surface of the virus, the haemagglutinin (HA) and the neuraminidase (NA). The HA is responsible for attachment and entry to host cells via sialic acid on cell receptors, whereas the NA is an enzyme that facilitates budding of new viral particles from the host cells by cleavage of the sialic acid-containing receptors. The M2 ion channel, which spans the viral membrane and is also exposed on the surface of the virus, is involved in proton conductance and is critical for replication. Influenza viruses are classified into three types, A, B, and C, according to antigenic differences between their NP and M proteins [3]. Based on antigenic variation, influenza A viruses are further divided into subtypes (e.g., A(H3N2)) based on the combination of their HA (H1-H18) and NA (N1-N9) proteins.

### 1.2. Viral Evolution and Pandemics

The continued spread of influenza virus amongst humans relies on antigenic variation of the HA and NA surface proteins, resulting from antigenic drift or antigenic shift. Antigenic drift is an accumulation of point mutations caused by inadequate proofreading by the RNA-dependent polymerases [4] that results in antigenic changes that allow the virus to escape the immune response, leading to recurrent seasonal influenza epidemics [1]. Mutations in the HA are primarily responsible for antigenic change, while mutations in the NA can result in changes in the shape of the NA enzymatic site, the target of the NA inhibitor antivirals, potentially resulting in drug resistance. Antigenic shift is the outcome of reassortment of the segmented viral genome, which can occur when a cell is simultaneously infected with two different influenza viruses [5]. The resulting virus may possess novel surface glycoproteins that are antigenically distinct from the currently circulating strains. Due to the lack of human population immunity, such novel variants can rapidly spread around the globe and cause a pandemic (worldwide epidemic) as demonstrated in 1918 (Spanish flu), 1957 (Asian flu), 1968 (Hong Kong flu), and most recently in 2009.

While influenza is typically considered a human disease, the natural reservoir of influenza A viruses exists in wild aquatic birds [6]. On occasions, influenza A viruses can cross the species barrier from birds to humans either directly or via an intermediary host such as pigs. In the last decade, there have been many cases of zoonotic transmission of influenza viruses from either avian or swine sources into humans. Human infections of swine-origin variant A(H3N2)v, A(H1N1)v, and A(H1N2)v viruses in the United States of America (USA) have typically caused only mild disease [7], while other more virulent avian-origin viruses such as A(H5N1) and A(H7N9) have caused severe disease and a high case fatality rate [8, 9]. Although these influenza viruses can cause severe disease in humans, they have not yet developed the capacity to transmit efficiently between humans. However, in 2009, a highly transmissible swine influenza virus, which initially infected humans in Mexico, subsequently spread globally resulting in the first influenza pandemic of the 21st century. The virus, known as A(H1N1)pdm09, continues to circulate in humans together with A(H3N2) viruses and influenza B viruses and is now considered a “seasonal” influenza strain.

### 1.3. Control and Management of Influenza

Vaccination is currently the most effective method for preventing influenza infection. Large quantities of seasonal influenza vaccines are used each year, predominantly in developed countries and particularly for elderly patients. Because of antigenic drift, the World Health Organization (WHO) Global Influenza Surveillance and Response System (GISRS), a network of laboratories across 111 WHO Member States, characterizes circulating viruses and isolates candidate vaccine viruses to ensure that the vaccines remain antigenically relevant [10].

Antivirals play an important role in the prevention and treatment of influenza infections in pandemic situations but for seasonal influenza are predominantly used for the treatment of influenza in severely ill patients including immunocompromised individuals [11]. In addition, the use of antivirals for short-term prophylaxis to prevent the spread of influenza within households [12] or closed institutional settings such as nursing homes or military camps has been well demonstrated [13, 14]. As part of pandemic preparedness plans, many countries have stockpiled influenza antivirals for the use in the early period of a pandemic when strain-specific vaccines are unavailable.

### 1.4. Influenza Antivirals: Adamantanes and Neuraminidase Inhibitors

There are currently two classes of influenza antivirals approved for use in many countries—the adamantanes and the neuraminidase inhibitors (NAIs). The adamantanes, specifically amantadine (Symmetrel) and rimantadine (Flumadine), were the first influenza antivirals to be approved for the treatment and prophylaxis of influenza [15]. The adamantanes inhibit viral replication by blocking the activity of the M2 ion channel that is essential for viral uncoating following entry into the cell [16]. However, because of structural differences between influenza A and B viruses, these drugs are not effective against influenza B viruses [17]. Clinical studies have shown that adamantanes have a 70–90% prophylactic efficacy, as well as a therapeutic benefit [18]. However, the largest problem with adamantane therapy has been the rapid selection of drug-resistant viruses that can spread efficiently in the presence or absence of the drug.

The NAIs are a class of anti-influenza drugs that bind to the enzymatic site of the neuraminidase glycoprotein of influenza A and B viruses, inhibiting the normal function of the neuraminidase, thereby preventing the release of viral progeny from infected cells [15]. The NAIs zanamivir (Relenza) and oseltamivir (Tamiflu) were approved across many countries in 1999-2000, with other newer NAIs such as laninamivir and peramivir approved in Japan, South Korea, and China since 2010 [19, 20]. Although all of the NAIs bind to the active site of the NA, they differ slightly in their chemical structure and binding characteristics. In addition, their typical mode of delivery differs: oseltamivir is delivered orally, zanamivir and laninamivir are delivered by inhalation, and peramivir is approved for intravenous delivery [20]. To date, oseltamivir remains the most prescribed influenza antiviral, driven mainly by its ease of administration, with sales exceeding those of zanamivir by at least 10-fold [21]. The majority of the global use of NAIs has occurred in Japan, followed by the USA, and then the rest of the world [22]. In Japan, the use of laninamivir (a long-acting NAI which requires only a single administration that provides antiviral activity for 5–7 days) has recently surpassed that of oseltamivir, which requires a regimen of twice daily administration over a five-day period [23]. The NAIs have shown good efficacy for the treatment and prophylaxis of influenza A infection [24, 25], although a recent review of the literature suggests that the effectiveness of oseltamivir may be lower for the treatment of influenza B [26]. As with any antimicrobial, the development of resistance against the adamantanes and NAIs can mean that their therapeutic benefits are reduced or even abrogated.

## 2. Antiviral Susceptibility of Circulating Human Influenza Viruses

### 2.1. Overview of Antiviral Susceptibility Monitoring

As previously described, the global surveillance of influenza viruses is coordinated by the WHO GISRS. Influenza viruses are collected by laboratories in various regions throughout the world and sent to one of five WHO Collaborating Centres for Reference and Research on Influenza (WHO CC) where their antigenic, genetic, and antiviral susceptibility properties are fully characterized. Monitoring of circulating strains for antiviral susceptibility, together with related research on antiviral resistance, began in the WHO CC in Melbourne in the early 2000s.

### 2.2. Testing Methodologies

Molecular-based assays such as Sanger sequencing, real-time RT-PCR, and pyrosequencing are well suited to monitor adamantane susceptibility, because resistance is associated with only five amino acid residues in the M2 protein (residues 26, 27, 30, 31, and 34). In contrast, a considerably larger number of mutations have been shown to alter NAI susceptibility [27]. Therefore, the use of a functional assay that measures the drug concentration required to inhibit 50% of the viral NA enzyme activity (IC_50_) is considered the “gold-standard” methodology for determining NAI susceptibility. Two similar enzyme inhibition assays, a chemiluminescent-based (CL) [28] or a fluorescent-based (FL) assay [29], are typically used. The FL assay is less costly to perform and better at discriminating between NAI susceptible and resistant viruses, while the CL assay typically provides improved signal to noise linearity and higher sensitivity in measuring NA activity [30]. For laboratories that choose not to establish the NA enzyme inhibition assay, molecular-based assays that target the most commonly detected mutations that alter NAI susceptibility can be utilized. However, it is important to be aware that the absence of common resistance mutations does not indicate that the viruses are NAI-sensitive, as novel mutations may occur. An alternative to the FL and CL assays is a bioluminescence-based NA inhibition assay (QFlu) that has the main advantage of being sensitive enough to detect low levels of virus in clinical specimens compared to the FL and CL assay which require a cultured viral isolate [31].

### 2.3. Adamantane Resistance in Circulating Viruses

**Figure 1:**
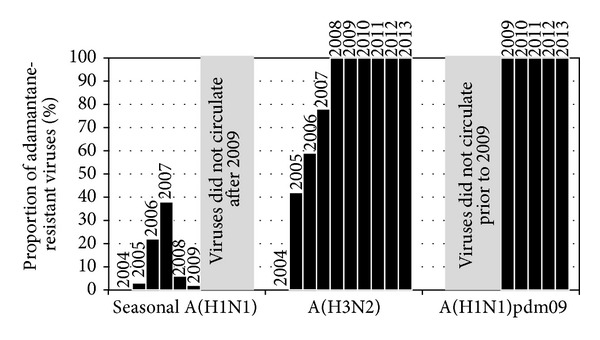
Frequency of adamantane-resistant viruses circulating in the Asia-Pacific.

In 2003, adamantane-resistant A(H3N2) viruses first began circulating in South Korea, Taiwan, Hong Kong, and China at frequencies ranging from 15 to 74% [32]. However, adamantane-resistant viruses were not detected in Australasia and neighboring Asian-Pacific countries until 2005, when 42% (43/102) of A(H3N2) isolates were resistant [33] (Figure 1). Such findings were concerning as many countries, including Australia, prescribed very little if any adamantanes, suggesting that these resistant viruses were spreading in the absence of drug pressure. The proportion of the adamantane-resistant A(H3N2) viruses circulating in the Asia-Pacific continued to increase in subsequent years (59% in 2006 and 78% in 2007) until it reached 100% in 2008 (Figure 1). Since that time all A(H3N2) viruses have remained adamantane-resistant, due to the S31N M2 gene mutation.

Remarkably, at the same time that A(H3N2) adamantane-resistant viruses began to emerge, adamantane-resistant seasonal A(H1N1) viruses also began circulating in the USA [34] and other parts of the world, including the Asia-Pacific [35, 36]. Of the seasonal A(H1N1) samples tested at the WHO CC in Melbourne, the frequency that were adamantane-resistant (again due predominantly to the S31N M2 gene substitution) rose from 3% in 2005 to 38% in 2007, before dropping considerably in 2008 and 2009 (Figure 1). The adamantane-resistant A(H1N1) viruses were within two genetically distinct groups of seasonal A(H1N1) viruses known as Clade 2A and 2C (Hong-Kong-like lineage) that were circulating between 2005 and 2008. However, in 2008, a new group of antigenic variants (known as Clade 2B [A/Brisbane/59/2007-like lineage]) emerged and began to replace the previously circulating Hong-Kong-like viruses. Compared to its predecessor, this new antigenic variant was adamantane-sensitive, thus reducing the overall frequency of adamantane-resistant A(H1N1) viruses (Figure 1).

Since the emergence of A(H1N1)pdm09 in 2009, the seasonal A(H1N1) viruses have become extinct. The A(H1N1)pdm09 pandemic virus already contained the M2 gene from Eurasian swine-lineage viruses which had the S31N substitution that confers adamantane-resistance. As a result, the A(H1N1)pdm09 viruses were, and continue to be, resistant to the adamantanes (Figure 1) [37].

### 2.4. NAI Resistance in Human Influenza Viruses (Pre-2009 Pandemic)

In addition to the antiviral susceptibility testing within the WHO GISRS, the Neuraminidase Inhibitor Susceptibility Network coordinated testing of circulating influenza viruses for NAI sensitivity before and after market release of the NAIs. Of the viruses circulating during the three years preceding the introduction of the NAIs (1996 to 1999), none was found to be resistant [38], while analysis of viruses postmarket release (1999 to 2002) found only a very low frequency of resistant viruses across all types or subtypes (<0.5%) [39]. Similar data were generated by our laboratory, showing that the vast majority of circulating human influenza viruses sampled around Australasia and Southeast Asia between 1998 and 2002 were sensitive to both oseltamivir and zanamivir [40]. Between the time of NAI market release and 2007, the situation remained much the same, with the detection of a very low (<1%) frequency of NAI-resistant viruses circulating in the community. Of the NAI-resistant viruses detected, the majority were oseltamivir-resistant, with very few showing any increase in zanamivir IC_50_. However, a small number of zanamivir-resistant seasonal A(H1N1) viruses with a Q136K NA mutation were detected by our laboratory and shown to have approximately 300-fold and 70-fold reduction in susceptibility to zanamivir and peramivir, respectively, but no loss of oseltamivir susceptibility [41]. Interestingly, the Q136K variants could not be detected in the original specimens, suggesting that the mutation arose during cell culture [41]. However, the zanamivir-resistant Q136K variants showed equivalent infectivity and transmissibility in a ferret model [41] and therefore may have the potential to emerge and spread amongst humans in the future.

In our laboratory between 2001 and 2005, we detected two oseltamivir-resistant viruses from otherwise healthy children who were not being treated with NAIs, suggesting that either the viruses were contracted from patients undergoing treatment or that the mutations had arisen in the absence of treatment. The first was an influenza B virus that contained a previously unreported NA mutation (D197E) [42–44], while the other was a seasonal A(H1N1) virus with an H275Y NA mutation that was resistant to oseltamivir (approximately 900-fold higher IC_50_ compared to a sensitive wild-type virus) but retained sensitivity to zanamivir [45]. Other studies had also detected the H275Y-variant A(H1N1) virus in patients undergoing oseltamivir treatment [46, 47] and in untreated patients from the community [39], demonstrating the potential for this variant to arise under oseltamivir selective pressure and transmit.* In vitro* and* in vivo* studies showed that, in some cases, the seasonal A(H1N1) H275Y variants had compromised growth [47], while other studies showed in ferrets that replication and transmission of the H275Y variant were possible [48, 49].

**Figure 2:**
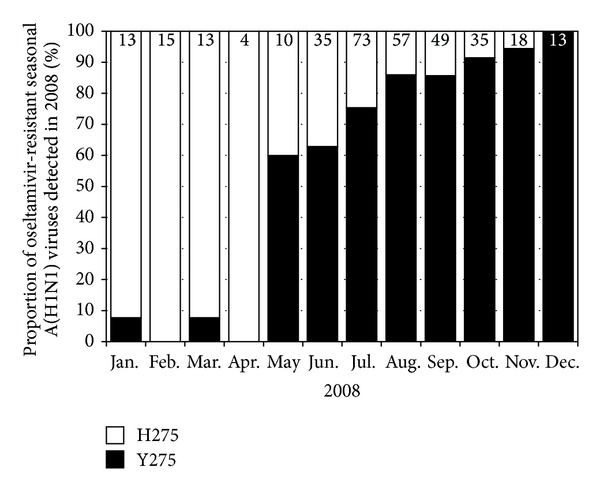
Proportion of oseltamivir-resistant seasonal A(H1N1) variants detected in the Asia-Pacific region during 2008. H275: oseltamivir-sensitive viruses and Y275: oseltamivir-resistant viruses. The number of viruses tested from each month is indicated at the top of each bar.

In late 2007, seasonal A(H1N1) viruses with the H275Y NA mutation began to circulate at an increasing frequency in Europe [50]. Compared to the normal frequency of the H275Y variant (<1%), many European countries detected the mutation in >20% of their circulating seasonal A(H1N1) viruses, with France and Norway detecting resistance at a frequency of >40% (231/496 (47%) and 184/272 (68%), resp.) [50]. It was particularly concerning that these resistant viruses were being detected in individuals not undergoing oseltamivir treatment, demonstrating that the viruses were spreading efficiently in a manner similar to that seen with adamantane-resistant viruses. The viruses continued to spread to the USA [51] and then to the Southern Hemisphere from May 2008 onwards [52, 53]. The H275Y-resistant viruses not only spread rapidly but also outcompeted the older oseltamivir-sensitive seasonal A(H1N1) viruses, so that, by the end of the 2008 Southern Hemisphere influenza season virtually all seasonal A(H1N1) viruses received at the WHO CC in Melbourne were oseltamivir-resistant [52]. This was therefore a transition from virtually no resistance to a frequency of >99% oseltamivir resistance in approximately 12 months (Figure 2). However, just as the world was coming to terms with the ongoing circulation of an oseltamivir-resistant virus, the A(H1N1)pdm09 pandemic virus emerged, outcompeting the oseltamivir-resistant seasonal A(H1N1) virus from circulation and providing a “beneficial” outcome of the 2009 pandemic.

### 2.5. NAI Resistance in Human Influenza Viruses (Post-2009 Pandemic)

During the 2009 pandemic, large volumes of NAIs, particularly oseltamivir, were used for the treatment and prophylaxis of patients prior to the availability of a pandemic vaccine [54]. Due to the earlier experience of the emergence and spread of oseltamivir-resistant seasonal A(H1N1) viruses with an H275Y NA mutation, there was great concern that A(H1N1)pdm09 viruses could also rapidly acquire this same mutation and spread, particularly in the face of increased NAI use. During the first year of the pandemic, global surveillance data generated within the WHO GISRS showed that the frequency of NAI resistance in >27,000 A(H1N1)pdm09 viruses was encouragingly low (*≈*1%) [11]. From the data derived by our laboratory following analysis of >2,900 A(H1N1)pdm09 viruses from the Asia-Pacific region, we showed a similarly low frequency (0.8 to 1.1%) of NAI resistance [55]. However, of the resistant viruses that were detected, virtually all contained the same H275Y NA mutation that was present in the previous oseltamivir-resistant seasonal A(H1N1) virus [11, 55]. Although unlike the seasonal A(H1N1) H275Y variants, the majority of A(H1N1)pdm09 H275Y variants were isolated from immunocompromised patients undergoing oseltamivir treatment for prolonged periods [55–58]. Nevertheless, it remained important to continue to monitor viruses from untreated patients in the community to determine if resistant viruses were spreading.

**Figure 3:**
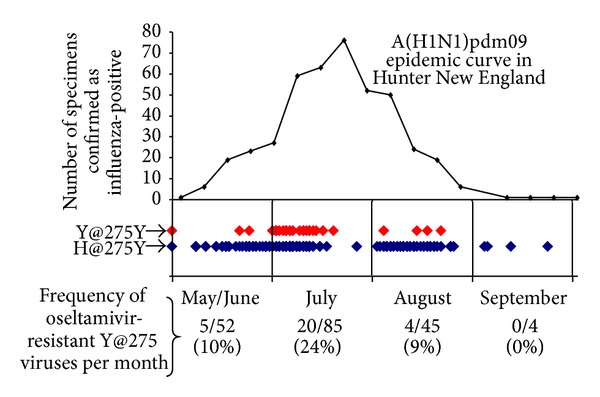
Frequency of oseltamivir-resistant A(H1N1)pdm09 viruses (Y@275) compared to oseltamivir-sensitive viruses (H@275) in the Hunter New England region of Australia in 2011.

While there had been a small number of reports of transmission of A(H1N1)pdm09 H275Y variants in closed, near-contact settings [59], such as hospital wards [60, 61], it was not until 2011 that a widespread cluster of related cases was observed in a community setting [62]. As a result of close collaboration between epidemiologists, the local diagnostic laboratory, and our WHO CC laboratory, we were able to detect this cluster of resistant viruses in the Hunter New England region around Newcastle, Australia. The rapid response enabled an increased testing of patients in the area and epidemiological follow-up of patients when a resistant virus was detected [62, 63]. Overall, 29/186 (16%) A(H1N1)pm09 viruses tested from the region were found to be oseltamivir-resistant, with resistance peaking (24%) in July (Figure 3). While resistant viruses related to the Newcastle cluster were detected in other parts of Australia [63, 64], fortunately, they did not spread internationally. However, reports from the UK [65], Netherlands [66], USA [67], and the Asia-Pacific [37] indicted an overall increase in the detection of oseltamivir-resistant viruses from untreated community patients around 2011, suggesting that A(H1N1)pdm09 H275Y variants may be becoming more capable of community transmission.

A(H1N1)pdm09 H275Y variants remain by far the most commonly detected NAI-resistant virus across all circulating types or subtypes. However, other mutations in the N1 of A(H1N1)pdm09 viruses such as S247N, I117V, and I223R have been shown to confer mild increases in IC_50_, but, when present in combination with the H275Y NA mutation, they substantially increase resistance above that observed for the H275Y mutation alone. A(H1N1)pdm09 viruses with S274N NA mutations were detected by our laboratory in two small clusters in Singapore and Australia [56]. A variant virus that contained the H275Y and S274N NA mutations was highly resistant to oseltamivir with an IC_50_ value nearly 6,000-fold greater than that seen for sensitive viruses [56]. A similar situation was observed for other variants with dual I117V + H275Y or I223R + H275Y mutations, where the oseltamivir IC_50_ was >2,000-fold and >9,000-fold higher than sensitive viruses, respectively, and significantly higher than viruses with the H275Y NA mutation alone [68, 69].

While the number of oseltamivir-resistant A(H1N1)pdm09 viruses has vastly exceeded that of NAI-resistant A(H3N2) or B viruses since 2009, the potential of these other subtypes to develop resistance should not be disregarded. The most commonly detected NA mutation in A(H3N2) oseltamivir-resistant viruses is E119V, which we recently reported in samples from an immunocompromised patient undergoing oseltamivir treatment [70]. Previous studies have shown that this resistant virus has the ability to transmit readily in an animal model and therefore may have the capacity for community spread [49]. Influenza B variants with some novel NA mutations have also been detected recently in our monitoring of circulating human influenza viruses [37].

## 3. Antiviral Susceptibility of Highly Pathogenic A(H5N1)avian Influenza Viruses

### 3.1. Susceptibility Monitoring and* In Vitro* Selection of NAI Resistance

In addition to the susceptibility monitoring of circulating human influenza viruses, our laboratory and others have also assessed the NAI and adamantane susceptibility of potential pandemic viruses such as highly pathogenic A(H5N1) viruses. We showed that 49/55 (89%) highly pathogenic A(H5N1) viruses from Vietnam, Indonesia, Cambodia, Myanmar, and Malaysia collected between 2004 and 2006 were resistant to the adamantanes, due to either S31N or dual S31N/L26I mutations in the M2 gene [71]. However, all but two of these viruses were sensitive to NAIs. One variant strain from Indonesia contained an I117V NA mutation which conferred a 16-fold increase in oseltamivir IC_50_ but no difference in zanamivir and peramivir sensitivity, while the other variant had a V116A NA mutation which conferred a 11-, 63-, and 4-fold increase in oseltamivir, zanamivir, and peramivir IC_50_, respectively, [71]. More recent analyses of highly pathogenic H5N1 viruses told a similar story, with a continued high frequency of adamantane resistance and a low frequency of NAI resistance [72, 73].

In addition to the monitoring of circulating A(H5N1) viruses for NAI sensitivity, we have conducted* in vitro* experiments to select NAI-resistant A(H5N1) variants under drug pressure. Under oseltamivir pressure, we selected variant viruses with H275Y, I223M, or dual H275Y + I223M NA mutations [74]. The H275Y A(H5N1) variant viruses had a 900- to 2500-fold reduction in oseltamivir susceptibility, whereas I222M variant viruses had a modest 36-fold reduction in oseltamivir susceptibility. However, dual H275Y + I222M NA mutations resulted in an extremely large reduction in oseltamivir susceptibility (~8000-fold) compared to wild-type viruses [74]. Under zanamivir selective pressure, A(H5N1) viruses with D198G and E119G NA mutations were selected. A D198G NA mutation conferred a 44-fold reduction in zanamivir susceptibility and a 32-fold reduction in oseltamivir susceptibility, while E119G conferred a large ~1500-fold reduction in zanamivir susceptibility but had little effect on oseltamivir sensitivity* in vitro* [74].

### 3.2. Assessing the Effectiveness of Peramivir and Oseltamivir Treatment against A(H5N1) in the Ferret Model

Ferrets are the preferred animal model to assess influenza virus infection, virulence, and transmission as they display similar clinical symptoms, pathogenesis, and antibody responses to those of humans [75]. In addition, ferrets can be readily infected with either seasonal, pandemic, or potentially pandemic influenza subtypes such as A(H1N1)pdm09, H7N9, and H5N1 [76–80]. We used the ferret model to assess the effectiveness of intramuscular injected peramivir and orally administered oseltamivir against highly pathogenic A(H5N1) viruses [81, 82]. Peramivir significantly improved survival over a 16–18-day period compared to untreated animals and reduced disease and viral load in the lung and brain of infected ferrets [81]. Oseltamivir was administered twice daily to ferrets infected with A(H5N1) A/Vietnam/1203/2004 at a range of different doses (0.1, 0.5, 2.5, and 5.0 mg/kg). Ferrets given 5 mg/kg oseltamivir survived A(H5N1) infection, while lower concentrations were unable to prevent mortality in animals [82].

## 4. Understanding and Assessing the Fitness of NAI-Resistant Viruses

Mutations in the NA that cause NAI resistance can alter enzyme activity or affinity for the natural substrate potentially compromising viral replication and transmission, or viral “fitness.” Therefore, when NAI-resistant viruses are detected, it is important to assess various* in vitro* and* in vivo* functional characteristics to determine the likelihood that a strain may spread. For example, the R292K NA substitution in A(H3N2) viruses causes an extremely large increase in oseltamivir IC_50_ but results in a virus that is unable to transmit between cohoused ferrets [83, 84]. In addition to describing the fitness of viruses detected through our surveillance program, our laboratory also uses site-directed mutagenesis and reverse genetics to “custom-make” influenza viruses with substitutions in NA amino acid residues of interest [84]. Conserved framework and catalytic residues of the N1 NA, E227, and E276, which had not previously been associated with NAI resistance, were modified (E227D, G, and V and E276D, Q, and K) to investigate their role in NA function and NAI sensitivity [85]. Of the different variants generated, only two (E227D and E276D) were able to replicate efficiently, and all had large reductions in NA activity [85]. Only the E227D virus demonstrated a significant reduction in sensitivity to the NAIs (126-fold reduction in zanamivir sensitivity), while E276D showed no reduction in NAI sensitivity. Such studies are useful in determining and/or confirming the role of individual residues in NA function, viral fitness, and NAI susceptibility.

*In vitro *assays that assess the NA enzyme activity, NA affinity, NA protein expression, and viral replication kinetics have been used to help understand the fitness costs of the seasonal A(H1N1) H275Y variant [86], as well as many other NAI-resistant viruses [87]. However,* in vivo* infectivity and transmissibility experiments in an animal model are needed to get a more complete picture of the likelihood that these viruses may spread to the human population. Ferret experiments have traditionally determined the minimum infectivity titre, duration, and peak titre of viral replication and transmission efficiency (number of animals infected/number of animals exposed) to assess the fitness of NAI-resistant viruses [41, 88]. However, in an attempt to detect subtle differences between the fitness of NAI-resistant virus and its respective NAI-sensitive virus, we developed a novel experimental ferret model known as “competitive mixtures” [83].

**Figure 4:**
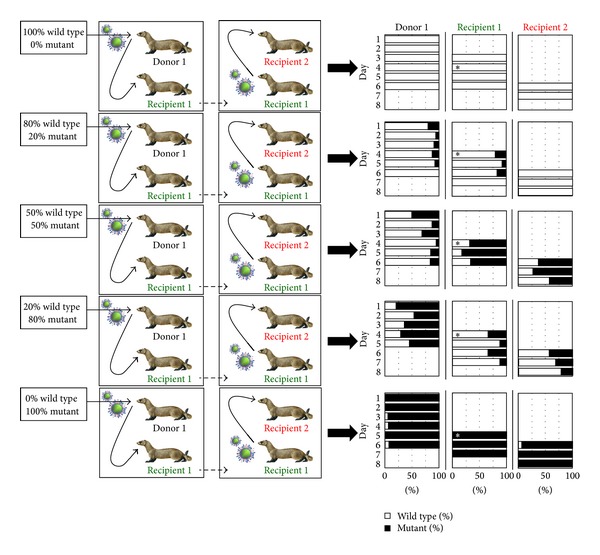
Overview of the competitive mixtures ferret model developed in our laboratory for assessing the relative fitness of a mutant virus compared to the wild type.

The competitive mixtures model involves the infection of ferrets with a mixture of two different viruses (e.g., a wild-type (sensitive) and mutant (NAI-resistant) virus) and the daily measurement of the relative proportion of those viruses to determine if one virus is replicating faster than the other. Typically, we perform experiments that involve the infection of “donor” ferrets with either a mixture (e.g., 80% : 20%, 50% : 50%, and 20% : 80%) or the pure virus (% based on a virus tissue culture infectivity titre), then, after 24 hours, the infected donor is cohoused with a naive ferret (recipient 1) (Figure 4). Once recipient 1 becomes infected, it is then cohoused with another naïve ferret (recipient 2) (Figure 4). All ferrets are nasal washed daily and samples analysed by pyrosequencing to test whether the proportion of the two viruses is changing within a ferret over the course of the infection or upon contact transmission between ferrets (Figure 4). The data can be inserted into a mathematical model to obtain a quantitative estimate of both the “within-host” (replication) and “between-host” (transmission) fitness of the mutant virus (compared to the wild type). We believe that the combination of the competitive mixtures ferret model together with the subsequent mathematical analysis makes this a highly informative and accurate experiment to assess the fitness of influenza viruses.

## 5. Conclusions and Future Directions

With currently circulating influenza viruses all showing resistance to the adamantanes, we rely exclusively on the NAIs for treatment and prophylaxis against influenza. Generally, little resistance has been observed against the NAIs, although there is the ongoing concern that oseltamivir-resistant A(H1N1)pdm09 H275Y variants may emerge. Our laboratory is currently engaged in studies to assess the permissive mutations that may be required in the A(H1N1)pdm09 virus that enable it to more easily acquire the H275Y NA mutation but retain viral fitness. New NAIs such as laninamivir and peramivir will become more widely used in the coming years. This would be welcome as a recent study by our laboratory showed that none of the viruses received at the WHO CC in Melbourne between 2009 and 2012 showed any resistance to the newly launched laninamivir [89], although a small number of viruses did show peramivir resistance, including the H275Y A(H1N1)pdm09 variants [89]. The cross-resistance seen between oseltamivir and peramivir is predominantly due to the hydrophobic pentyl side group that both structures share, but the implication of this is that peramivir is unlikely to be a good alternative for use against oseltamivir-resistant viruses.

In recent years, there has been a large increase in the different classes of influenza antivirals that have entered the clinical pipeline, including monoclonal antibodies, immunomodulatory agents, and receptor antagonists [90]. Currently, there are two influenza antivirals in phase III clinical trials, Nitazoxanide and Favipiravir, which target HA maturation and the polymerase, respectively. These antivirals and others offer the potential for combination therapy with the NAIs, which will hopefully improve the effectiveness of drug treatment and patient outcome as well as reducing the chances of developing drug resistance. Understanding more about how these new non-NAI antivirals work, their potential for development of resistance, and methodologies to enable high-throughput susceptibility analysis will be the focus of research efforts within our laboratory and others in the coming years.

Future studies will also involve the continued refinement of animal models to assess antiviral effectiveness. Experiments to compare how different antiviral treatment and prophylaxis regimens alter infection of contacts, viral kinetics, and clinical symptoms will help inform guidelines on the appropriate use of the drugs. In addition, such animal models can be used to provide insight into how* in vitro *IC_50_ data correlates with* in vivo* clinical efficacy. Without this link, it is challenging for clinicians to interpret IC_50_ data in a manner that allows them to make the most informed and appropriate choice of drug to be used on a patient.

While influenza viruses will continue to evolve, providing the potential for the emergence of variant or novel viruses into the human population, it will remain important for the WHO GISRS to maintain and hopefully expand its surveillance across the globe, to ensure that antigenic variants and antiviral-resistant viruses are detected as quickly as possible. The development of new antivirals as well as research into more broadly reactive influenza vaccines [91] will hopefully mean that, in the future, we can better manage the impact of influenza on the human population.
